# Whole Genome Sequencing Reveals a De Novo *SHANK3* Mutation in Familial Autism Spectrum Disorder

**DOI:** 10.1371/journal.pone.0116358

**Published:** 2015-02-03

**Authors:** Sergio I. Nemirovsky, Marta Córdoba, Jonathan J. Zaiat, Sabrina P. Completa, Patricia A. Vega, Dolores González-Morón, Nancy M. Medina, Mónica Fabbro, Soledad Romero, Bianca Brun, Santiago Revale, María Florencia Ogara, Adali Pecci, Marcelo Marti, Martin Vazquez, Adrián Turjanski, Marcelo A. Kauffman

**Affiliations:** 1 Plataforma de Bioinformática Argentina, Instituto de Cálculo, Pabellón 2, Ciudad Universitaria, Facultad de Ciencias Exactas y Naturales, UBA, Buenos Aires, Argentina; 2 Instituto de Agrobiotecnología de Rosario (INDEAR), CONICET, Predio CCT, Rosario, Argentina; 3 Departamento de Química Biológica, Pabellón 2, Ciudad Universitaria, Facultad de Ciencias Exactas y Naturales, UBA, INQUIMAE/CONICET, Buenos Aires, Argentina; 4 Consultorio y Laboratorio de Neurogenética. Hospital JM Ramos Mejía. IBCN Eduardo de Robertis UBA-CONICET, Buenos Aires, Argentina; 5 Departamento de Química Biológica, Facultad de Ciencias Exactas y Naturales, Universidad de Buenos Aires, IFIBYNE-CONICET, Facultad de Ciencias Exactas y Naturales, Universidad de Buenos Aires, Buenos Aires, Argentina; The George Washington University, UNITED STATES

## Abstract

**Introduction:**

Clinical genomics promise to be especially suitable for the study of etiologically heterogeneous conditions such as Autism Spectrum Disorder (ASD). Here we present three siblings with ASD where we evaluated the usefulness of Whole Genome Sequencing (WGS) for the diagnostic approach to ASD.

**Methods:**

We identified a family segregating ASD in three siblings with an unidentified cause. We performed WGS in the three probands and used a state-of-the-art comprehensive bioinformatic analysis pipeline and prioritized the identified variants located in genes likely to be related to ASD. We validated the finding by Sanger sequencing in the probands and their parents.

**Results:**

Three male siblings presented a syndrome characterized by severe intellectual disability, absence of language, autism spectrum symptoms and epilepsy with negative family history for mental retardation, language disorders, ASD or other psychiatric disorders. We found germline mosaicism for a heterozygous deletion of a cytosine in the exon 21 of the *SHANK3* gene, resulting in a missense sequence of 5 codons followed by a premature stop codon (NM_033517:c.3259_3259delC, p.Ser1088Profs*6).

**Conclusions:**

We reported an infrequent form of familial ASD where WGS proved useful in the clinic. We identified a mutation in *SHANK3* that underscores its relevance in Autism Spectrum Disorder.

## Introduction

Autism spectrum disorder (ASD) is a common cause of early disability, affecting about 1% of the population. It is characterized by impairments in social interaction and communication, as well as by repetitive and restricted behaviors [1].

ASD is etiologically heterogeneous, with hundreds of highly penetrant genetic aberrations involved as causative factors. Among them, *SHANK3* haploinsufficiency has been identified in about 0.5% of subjects with ASD. However, few familial ASD cases caused by mutations in *SHANK3* have been identified [2]. Here we present three siblings with ASD where Whole Genome Sequencing (WGS), identified germline mosaicism for a new mutation in *SHANK3*.

## Methods

### Study participants

The Ethics Committee of Hospital Ramos Mejía in Buenos Aires, Argentina approved this study. Written informed consents were obtained from the parents of the probands. Clinical investigation was conducted according to the principles expressed in the Declaration of Helsinki. The parents of the individuals described in this manuscript gave written informed consent to publish these case details. DNA sequencing data remained stored in a secure internal database, and is available upon request to researchers wishing to use them for research purposes only. Clinical evaluations were performed at the Neurogenetics Unit from Hospital Ramos Mejía.

### Genome sequence data generation

Genomic DNA was isolated from peripheral blood and sequenced using 101 base-pair paired-end reversible terminator massively parallel sequencing on an Illumina Hiseq 1500 instrument at INDEAR (Rosario, Argentina), following sequencing library preparation in agreement to standard Illumina protocols. Median fragment length of the libraries was 294 bp. A total of 334.1 Gb with quality equal or more than Q30 was produced for the three genomes. Specifically, 575,427,640 paired-end reads were produced for proband 1 (35.5X coverage), 603,002,568 paired-end reads were produced for proband 2 (37.18X coverage) and 527,754,644 paired-end reads were produced for proband 3 (32.58X coverage).

### Sequence Alignment and Annotation

Paired-end reads obtained from sequencing the probands’ whole genomes were aligned to the GRCh37 reference human genome using the Burrows-Wheeler Alignment Tool (BWA) [3]. The resulting SAM files were realigned and recalibrated by implementation of the Genome Analysis Toolkit (GATK) framework [4,5]. Variant calling was performed using the Unified Genotyper tools from GATK. Variant annotation and effect prediction was carried out with SnpEff 3.5a (build 2014-02-14) [6,7] with data from dbSNP Build 138 [8], the 1000 Genomes Project [9], the International HapMap Project [10], the NHGRI GWAS Catalog [11], dbNSFP v2.3 [12,13], the Exome Sequencing Project (ESP) [14], and ClinVar Build 20140211 [15]. See S1 Fig.

### Bioinformatic Prioritization of candidate variants (see Table 1)

Only high-quality variants (i.e. not filtered out by the GATK’s Variant Quality Score Recalibration method) were considered for the analysis. Those with recorded population frequencies estimates higher than 1% (data from the 1000 Genomes Project and the Exome Sequencing Project) were also discarded as unlikely to be of relevance. From this remaining set, variants with high probability of affecting gene function (frame-shift, nonsense, missense, affecting splice sites and small insertions and deletions) were grouped according to 3 different models of inheritance that may explain the probands’ phenotype: a recessive model grouped homozygous variants or compound heterozygous candidate variants present in the somatic chromosomes, a X-linked model grouped variants from the X chromosome, and a *high impact and rare variants* model grouped only high impact (nonsense, frame-shift, splice site changes) variants not recorded in the available databases. These groups were filtered through a list of 182 genes known to be of relevance in ASD according to SFARI database [16] (S1 Table).

**Table 1:** Summary of variants in the 3 genomes.

	**Proband 1 (III-1)**	**Proband 2 (III-2)**	**Proband 3 (III-3)**	**Shared**
**Reads**	1,150,855,280	1,206,005,136	1,055,509,288	-
**High Confidence (HC) Variants**	4,157,729	4,152,776	4,155,158	3,153,415
HC Variants in Coding Regions*	24,319	23,882	23,939	18,394
**HC Variants at Splice sites** **	467	450	447	351
**HC Single Nucleotide Variants (SNV)**	3,556,615	3,547,621	3,543,994	2,750,765
**HC Short Indels**	601,114	605,155	611,164	402,650
**HC Variants in ASD gene’s list**	71,348	69,173	69,215	53,358
**HC Variants compliant to the Recessive Model**	438	395	394	145
**Genes with HC variants compliant to the Recessive Model**	207	198	194	86
**HC Variants in in ASD genes and compliant with the Recessive Model**	2	1	1	1
**ASD genes with HC Variants compliant with the Recessive Model**	2	1	1	1
**HC Variants compliant to the X-Linked Model**	16	11	10	5
**Genes with HC variants compliant to the X-Linked Model**	14	10	9	4
**HC Variants in in ASD genes and compliant with the X-Linked Model**	1	0	0	0
**ASD genes with HC Variants compliant with the X-Linked Model**	1	0	0	0
**HC Variants compliant to the High Impact De Novo Model**	24	35	28	6
**Genes with HC variants compliant to the High Impact De Novo Model**	23	35	27	6
**HC Variants in ASD genes and compliant with the High Impact De Novo Model**	1	1	1	1
**ASD genes with HC Variants compliant with the High Impact De Novo Model**	1	1	1	1

* Including all changes withing coding regions (synonymous and nonsynonymous substitutions and short insertions and deletions)

** Defined as 2 bases after exon end or 2 bases before exon start (only measured in introns).

### Manual Review of Candidate variants

After prioritization, variants considered to possibly explain the phenotype were further investigated individually through the use of several databases and functional evaluation tools which included those provided by NCBI (www.ncbi.nlm.nih.gov), ENSEMBL [17] (www.ensembl.org/index.html), Mutation Taster [18] (www.mutationtaster.org), the Combined Annotation Dependent Depletion framework [19] (cadd.gs.washington.edu) and the Search Tool for the Retrieval of Interacting Genes/Proteins [20] (STRING; string-db.org), among others.

## Results

### Case Descriptions

**Figure 1:**
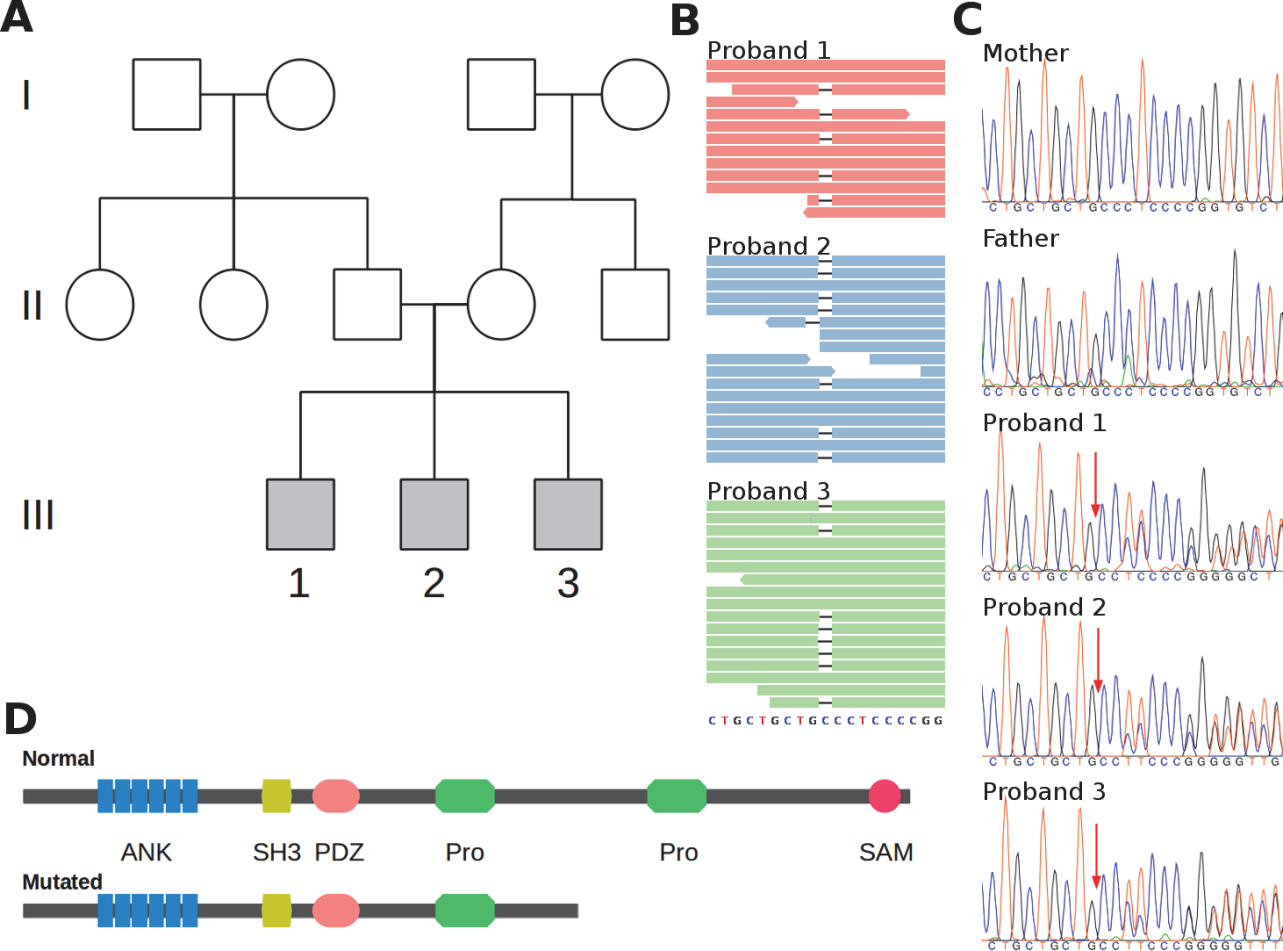
A SHANK3 point mutation in three siblings with Autism Spectrum Disorder. A) Family pedigree depicting the three probands (III-1, III-2, III-3), parents, their siblings and grandparents. B) Mutation as evidenced by whole genome sequencing compared to reference sequence (GRCh37) at bottom. Broad lines represent aligned reads. The heterozygous deletion is depicted as black, thin lines that interrupt the reads. Each panel depicts the data from one proband, C) Capillary sequencing chromatograms of the probands and their parents. A red arrow signals the position of the deletion. The change in ORF is evidenced by the presence of double peaks after the deletion site caused by heterozygocity. D) Linear representations of the intact *SHANK3* protein featuring its major domains and the presumptive protein if translated from the mutated sequence. ANK: ankyrin repeats, SH3: SRC Homology 3 domain, PDZ: PDZ domain, Pro: Proline-rich region, SAM: Sterile alpha motif domain.

Three male siblings were included in this study. All of them presented a syndrome characterized by severe intellectual disability, absence of language, autism spectrum symptoms and epilepsy. Family history was otherwise negative for mental retardation, language disorders, ASD or other psychiatric disorders. They were the sole offspring of healthy and unrelated parents after full-term and uneventful pregnancies (Fig. 1A). The mother performed appropriate health checks during gestations and she didn’t show any pathological findings. Deliveries and birth parameters were unremarkable. The eldest proband (III-1 in Fig. 1A) was symptomatic since birth. Neonatal hypotonia was manifested by sucking difficulties and a mild delay in motor development. He had limited eye contact since 6 months of age. He never developed a functional language. He showed severe behavioral problems, motor stereotypes and circumscribed interests since the age of four. He developed epilepsy at the age of 6, suffering from frequent atonic seizure non-respondent to classical antiepileptic drugs. The younger siblings (III-2 and III-3) showed very similar clinical features. They shared dysmorphic features: broad nasal bridge, bulbous nasal root and macrostomia. They exhibited no neonatal hypotonia and showed normal development during their first two years of life with no observable alterations in language. A progressive deterioration started after this age. Language development was arrested, evolving to an almost complete absence of verbal communication. Social interaction became severely impaired. Agitation and repetitive motor behaviors were present since the age of three. Epilepsy with frequent atonic and generalized seizures refractory to medical treatment started at the age of 7 in both siblings. MRIs were normal and some of the several EEGs done showed right-temporal discharges. Standard karyotype and fragile X testing were normal in the three siblings.

### Whole Genome Analysis

We performed whole genome sequencing of DNA samples from the three siblings, obtaining a mappable yield of 334.1 Gb, which represent an average depth of coverage of 33.04x. We identified more than 4.1 millions of variants in each genome (Table 1). We examined those rare variants (population frequency lower than 1%) in 182 genes associated to ASD (S1 Table) under three possible models of inheritance: *recessive, X-linked, high impact de novo mutations (S2 Table, S3 Table)*. The most plausible candidate to explain the phenotype exhibited by the probands was a heterozygous deletion of a cysteine in the exon 21 of the *SHANK3* gene (Fig. 1, B), resulting in a missense sequence of 5 codons followed by a premature stop codon (NM_033517:c.3259_3259delC, p.Ser1088Profs*6). We did not find any difference in *SHANK3* mRNA levels in blood among the three affected siblings, their parents and two unrelated controls, suggesting an absence of effect at the transcription level. Therefore, when expressed this protein would lack a Proline-rich region and the Sterile alpha motif (SAM) domain (Fig. 1, D) resulting in a functional haploinsufficiency of *SHANK*3 protein. Sanger sequencing confirmed this deletion in the three siblings and their absence in DNA purified from blood samples of both parents suggesting germinal mosaicism as the origin of the mutation (Fig. 1, C).

## Discussion

Gene mutations can be identified in about 20% of individuals with ASD [21]. *SHANK3* aberrations have consistently been associated with idiopathic and syndromic ASD. However, point mutations in *SHANK3* have been rarely reported. A few patients were previously described with only one showing familial recurrence. It has been suggested that nonsense and frameshift mutations in this gene lead to more severe phenotypes [22] affecting language and development as it was observed in our patients. Moreover, a recent and exhaustive report on the effect of different mutations in the SHANK family of genes in ASD concluded that *SHANK3* mutations are probably the most prevalent etiology of ASD with mental retardation and among the different cases described there it is noteworthy to highlight one that have a frameshift deletion predicted to result in a truncated protein lacking the same functional domains that we predict for our mutation, thereby supporting a pathogenic role for the variant here described, precluding the need for more thorough functional assays [23]. The product of this gene is expressed at postsynaptic densities of excitatory glutamatergic synapses and it is involved in synaptic maturation [24]. Moreover, pharmacological restoring of its deficiency is current object of therapeutic research in ASD [25].

The use of WGS in the clinic is called to be transformative, especially in a complex etiologically heterogeneous disorder such as ASD and intellectual disability [26]. However, recent studies have questioned its readiness for diagnostic adoption mostly because of uncertainties associated with the analysis of such vast amount of information [27]. We have ruled out many variants with a predicted high-impact on protein function because we opted to focus our analysis on genes considered to be of relevance for the phenotype studied therefore decreasing interpretation uncertainties. This left the reported *SHANK3* mutation as the sole variant putatively relevant to the phenotype showed in our patients. It is noteworthy to mention that by identifying the pathogenic variant causing the disorder segregating in this family, almost a decade of anxiety associated with diagnostic uncertainties could be alleviated. However, even though we could explain the recurrence of the disorder in the three siblings with the presence of germline mosaicism we could not estimate future recurrence risk because the proportion of germ cells mutated could not be quantified.

In summary, we reported an infrequent form of familial ASD where WGS proved useful in the clinic.

## Supporting Information

S1 Fig Flow diagram of the analysis. Reads resulting from Whole Genome Sequencing were aligned to the reference genome (GRCh37) with BWA and followed by realignment and recalibration with the Genome Analysis Toolkit (GATK). Variant calling was performed with the Unified Genotyper tool from the GATK, and annotated with SnpEff (see S1 Methods). After the whole variants set was produced, variants shared by the 3 probands were filtered if they presented population frequencies higher than 1% and according to the inheritance models described in Methods. These sets were then filtered by the ASD genes lists (see S1 Table) and manually reviewed for validation. Also, variants resulting from the first filter but not present after the second filter were manually screened and discarded if they showed no relation to the probands phenotype.
(TIF)

S1 Methods (DOCX)

S1 Table Candidate genes. (DOCX)

S2 Table Inheritance Models. (DOCX)

S3 Table High impact High confidence intersection Variants. (XLSX)
