# Supplementary figures and images for: Whole Genome Sequencing Reveals a De Novo SHANK3 Mutation in Familial Autism Spectrum Disorder
Source: PLoS One. 2015 Feb 3;10(2):e0116358. doi: 10.1371/journal.pone.0116358 (PMC4315573; doi:10.1371/journal.pone.0116358)

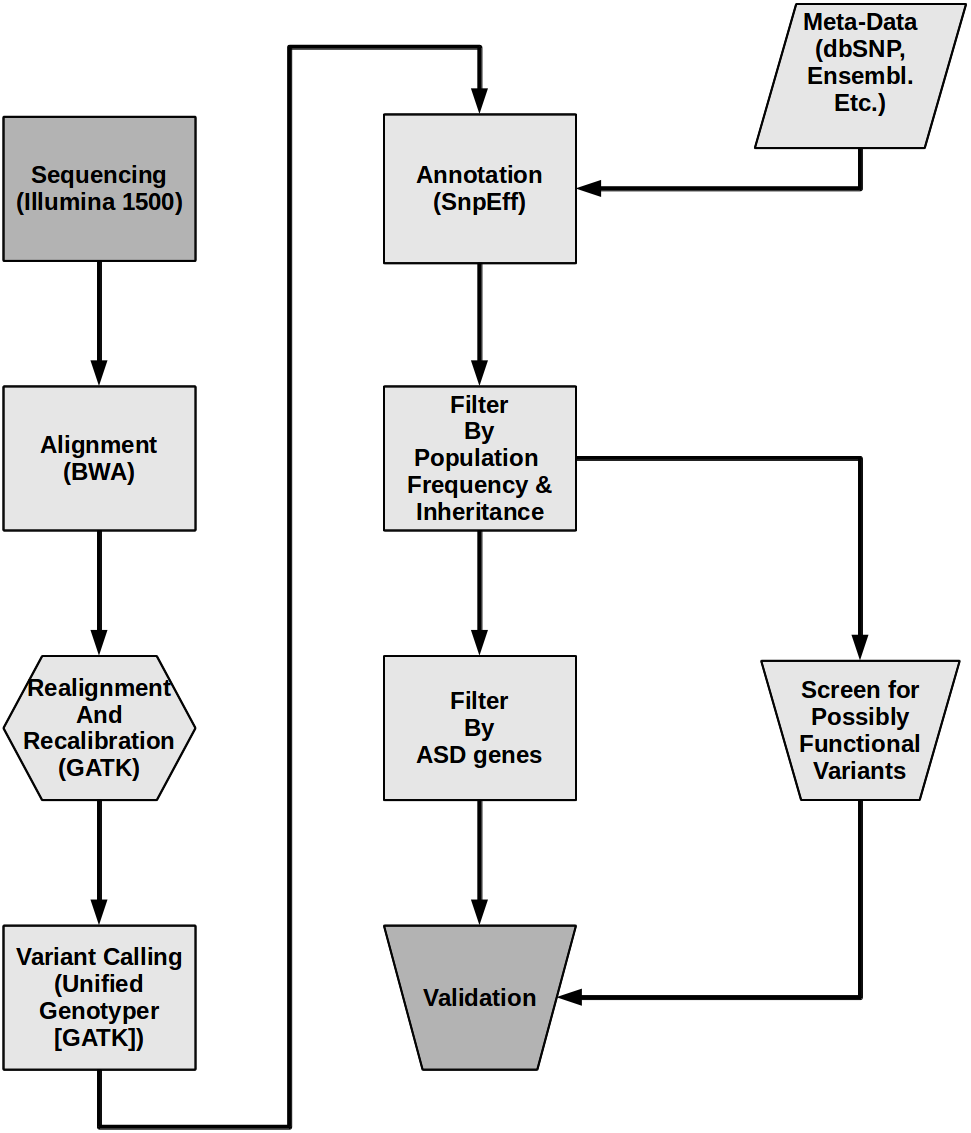

Supplement: S1 Fig — Reads resulting from Whole Genome Sequencing were aligned to the reference genome (GRCh37) with BWA and followed by realignment and recalibration with the Genome Analysis Toolkit (GATK). Variant calling was performed with the Unified Genotyper tool from the GATK, and annotated with SnpEff (see S1 Methods). After the whole variants set was produced, variants shared by the 3 probands were filtered if they presented population frequencies higher than 1% and according to the inheritance models described in Methods. These sets were then filtered by the ASD genes lists (see S1 Table) and manually reviewed for validation. Also, variants resulting from the first filter but not present after the second filter were manually screened and discarded if they showed no relation to the probands phenotype. (TIF) [file pone.0116358.s001.tif]
