# Supplementary material for: Whole Genome Sequencing Reveals a De Novo SHANK3 Mutation in Familial Autism Spectrum Disorder
Source: PLoS One. 2015 Feb 3;10(2):e0116358. doi: 10.1371/journal.pone.0116358 (PMC4315573; doi:10.1371/journal.pone.0116358)
Supplement: S1 Methods — (DOCX) [file pone.0116358.s002.docx]

# S1 Methods

# Manual Review of Candidate Variants

The manual review of candidate variants comprised those variants selected by the filtering methods and others found of high impact, which were not included in those groups. The filtering for this last group of variants was accomplished using the gene sets associated by ontologies of the Molecular Signatures Database of the Gene Enrichment Analysis website (http://www.broadinstitute.org/gsea/msigdb/index.jsp). A complete list of the ontologies used follows:

GO:0007218 NEUROPEPTIDE_SIGNALING_PATHWAY

GO:0007269 NEUROTRANSMITTER_SECRETION

GO:0007422 PERIPHERAL_NERVOUS_SYSTEM_DEVELOPMENT

GO:0006813 POTASSIUM_ION_TRANSPORT

GO:0001508 REGULATION_OF_ACTION_POTENTIAL

GO:0050770 REGULATION_OF_AXONOGENESIS

GO:0050767 REGULATION_OF_NEUROGENESIS

GO:0043523 REGULATION_OF_NEURON_APOPTOSIS

GO:0001505 REGULATION_OF_NEUROTRANSMITTER_LEVELS

GO:0007423 SENSORY_ORGAN_DEVELOPMENT

GO:0007600 SENSORY_PERCEPTION

GO:0050808 SYNAPSE_ORGANIZATION_AND_BIOGENESIS

GO:0007268 SYNAPTIC_TRANSMISSION

GO:0007416 SYNAPTOGENESIS

GO:0000041 TRANSITION_METAL_ION_TRANSPORT

GO:0019226 TRANSMISSION_OF_NERVE_IMPULSE

First assessment of each selected variant was accomplished through consultation of GeneCards database ([www.genecards.org](http://www.genecards.org/)) to learn more about the possibly affected gene. Usually the variants evaluated outside from the main filtering pipeline were homozygous. In the case of unknown variants (without dbSNP ID number) their impact was consulted through Mutation Taster ([www.mutationtaster.org](http://www.mutationtaster.org/)) in addition to the annotated scores (SIFT, phyloP, PhastCons and Mutation Assessor scores). Variants with dbSNP IDs and population frequencies of 1% (in 1000 genomes or EVS) or lower were probed, with the first step being consulting ENSEMBL's and dbSNP's population data. Variants were discarded in this manner if the happened to be present in the Venter or other reported genomes. Also, number of affected transcripts was considered when evaluating the effect.

# RNA isolation and RT-real time PCR

RNA was extracted from peripheral blood with the Trizol reagent according to the manufacturer instructions. For RT-real time PCR, 1.5 µg of total RNA was retrotranscribed with the MMLV enzyme (Promega), and an aliquot was used to real-time PCR. All reactions were conducted in a volume of 25 ul containing 4mM MgCl_2_ (Invitrogen), 0.25mM dNTPs (Invitrogen), 1.25U of Taq polymerase (Invitrogen), 1/30,000 Sybr Green (Roche, Indianapolis, IN), and specific oligonucleotides for each gene in a DNA Engine Opticon instrument (MJ Research Inc., Waltham, MA). Reactions were run for 40 cycles under the following conditions: 15 sec at 95 °C, 20 sec at 60 °C or 61 °C, and 25 sec at 72 °C. The amplification of unique products in each reaction was verified by melting curve and ethidium bromide (Sigma Aldrich) stained agarose gel electrophoresis. Each sample was run in triplicate. The expression level of each gene was normalized to TBP and β-actin expression level using standard curve method. The oligonucleotides used for each gene were as follows:

SHANK3 Fw GATGAGGCAGCATGACACAC

SHANK3 Rev GGACAGCCACTTTGTCATCA

TBP Fw TTCGGAGAGTTCTGGGATTG

TBP Rev AGCAAACCGCTTGGGATTAT

β-actin Fw CTCTTCCAGCCTTCCTTCCT

β-actin Rev AGCACTGTGTTGGCGTACAG

# Primers used for validation by Sanger sequencing of the SHANK3 mutation identified

Forward primer: TATCCCGAGCGGCAGAAG

Reverse primer: ACTGTGCACGGGTGTGG


PCR Product:

product length = 756

>gi|224589814|ref|NC_000022.10|:51158905-51159660 Homo sapiens chromosome 22, GRCh37.p13 Primary Assembly
TATCCCGAGCGGCAGAAGCGCGCGCGCTCCATGATCATCCTGCAGGACTCGGCGCCCGAGTCGGGCGACGCCCCTCGACCCCCGCCCGCGGCCACCCCGCCCGAGCGACCCAAGCGCCGGCCGCGGCCGCCCGGCCCCGACAGCCCCTACGCCAACCTGGGCGCCTTCAGCGCCAGCCTCTTCGCTCCGTCCAAGCCGCAGCGCCGCAAGAGCCCCCTGGTGAAGCAGCTGCAGGTGGAGGACGCGCAGGAGCGCGCGGCCCTGGCCGTGGGCAGCCCCGGTCCCGGCGGCGGCAGCTTCGCCCGCGAGCCCTCCCCGACCCACCGCGGTCCGCGCCCGGGTGGCCTCGACTACGGCGCGGGCGATGGCCCGGGGCTCGCGTTCGGCGGCCCGGGCCCGGCCAAGGACCGGCGGCTGGAGGAGCGGCGCCGCTCCACTGTGTTCCTGTCCGTGGGGGCCATCGAGGGCAGCGCCCCCGGC
GCGGATCTGCCATCCCTACAGCCCTCCCGCTCCATCGACGAGCGCCTCCTGGGGACCGGCCCCACCGCCGGCCGCGACCTGCTGCTGCCCTCCCCGGTGTCTGCCCTGAAGCCGTTGGTCAGCGGCCCGAGCCTGGGGCCCTCGGGTTCCACCTTCATCCACCCACTCACCGGCAAACCCCTGGACCCCAGCTCACCCCTGGCCCTTGCCCTGGCTGCCCGAGAGCGAGCTCTGGCCTCCCAGGCGCCCTCCCGGTCCCCCACACCCGTGCACAGT
