# Supplementary material for: Whole Genome Sequencing Reveals a De Novo SHANK3 Mutation in Familial Autism Spectrum Disorder
Source: PLoS One. 2015 Feb 3;10(2):e0116358. doi: 10.1371/journal.pone.0116358 (PMC4315573; doi:10.1371/journal.pone.0116358)
Supplement: S1 Table — (DOCX) [file pone.0116358.s003.docx]

**S1 TABLE**

**LIST OF CANDIDATE GENES FOR AUTISM SPECTRUM DISORDERS**

| GENE SYMBOL | GENE NAME |
| --- | --- |
| ADK | Adenosine kinase |
| ADNP | Activity-dependent neuroprotector homeobox |
| ADSL | Adenylosuccinate lyase |
| AFF2 | AF4/FMR2 family, member 2 |
| AFF4 | AF4/FMR2 family, member 4 |
| AGTR2 | Angiotensin II receptor, type 2 |
| AHI1 | Abelson helper integration site |
| ANK2 | Ankyrin 2, neuronal |
| ANKRD11 | Ankyrin repeat domain 11 |
| APBA2 | Amyloid beta (A4) precursor protein-binding, family A, member 2 |
| ARHGAP15 | Rho GTPase activating protein 15 |
| ARID1B | AT rich interactive domain 1B (SWI1-like) |
| ARX | A ristaless related homeobox |
| ASTN2 | Astrotactin 2 |
| ATP10A | Probable phospholipid-transporting ATPase VA |
| ATP2B2 | ATPase, Ca++ transporting, plasma membrane 2 |
| ATRX | Alpha thalassemia/mental retardation syndrome X-linked |
| AVPR1A | Arginine vasopressin receptor 1A |
| BAIAP2 | BAI1-associated protein 2 |
| BZRAP1 | Benzodiazapine receptor (peripheral) associated protein 1 |
| C15orf43 | Chromosome 15 open reading frame 43 |
| CA6 | Carbonic anhydrase VI |
| CADPS2 | Ca2+-dependent activator protein for secretion 2 |
| CAMTA1 | Calmodulin binding transcription activator 1 |
| CDH22 | Cadherin-like 22 |
| CDH8 | Cadherin 8, type 2 |
| CDKL5 | Cyclin-dependent kinase-like 5 |
| CEP41 | Testis specific, 14 |
| CHD7 | Chromodomain helicase DNA binding protein 7 |
| CHRNA7 | Cholinergic receptor, nicotinic, alpha 7 |
| CHST5 | Carbohydrate sulfotransferase 5 |
| CLTCL1 | Clathrin, heavy chain-like 1 |
| CNTNAP2 | Contactin associated protein-like 2 |
| CNTNAP5 | Contactin associated protein-like 5 |
| CSNK1D | Casein kinase 1, delta |
| CTNNA3 | Catenin (cadherin-associated protein), alpha 3 |
| CTNNB1 | Catenin (cadherin-associated protein), beta 1 |
| CUL3 | Cullin 3 |
| CXCR3 | Chemokine (C-X-C motif) receptor 3 |
| DAPK1 | death-associated protein kinase 1 |
| DCTN5 | dynactin 5 |
| DDX53 | DEAD (Asp-Glu-Ala-Asp) box polypeptide 53 |
| DHCR7 | 7-dehydrocholesterol reductase |
| DIAPH3 | Diaphanous-related formin 3 |
| DLX6 | Distal-less homeobox 6 |
| DPP10 | Dipeptidyl-peptidase 10 |
| DPYD | Dihydropyrimidine dehydrogenase |
| EGR2 | Early growth response 2 (Krox-20 homolog, Drosophila) |
| EHMT1 | Euchromatic histone-lysine N-methyltransferase 1 |
| EIF4E | Eukaryotic translation initiation factor 4E |
| EML1 | Echinoderm microtubule associated protein like 1 |
| EPC2 | Enhancer of polycomb homolog 2 (Drosophila) |
| EPHA6 | EPH receptor A6 |
| EPHB6 | EPH receptor B6 |
| ERBB4 | v-erb-a erythroblastic leukemia viral oncogene homolog 4 |
| EXT1 | Exostosin 1 |
| F13A1 | Coagulation factor XIII, A1 polypeptide |
| FBXO33 | F-box protein 33 |
| FBXO40 | F-box protein 40 |
| FGD1 | FYVE, RhoGEF and PH domain containing 1 |
| FGFBP3 | Fibroblast growth factor binding protein 3 |
| FOXG1 | Forkhead box G1 |
| FOXP1 | Forkhead box P1 |
| FOXP2 | Forkhead box P2 |
| FRMPD4 | FERM and PDZ domain containing 4 |
| GABRB3 | Gamma-aminobutyric acid (GABA) A receptor, beta 3 |
| GALNT13 | UDP-N-acetyl-alpha-D-galactosamine:polypeptide N-acetylgalactosaminyltransferase 13 (GalNAc-T13) |
| GLRA2 | Glycine receptor, alpha 2 |
| GNA14 | Guanine nucleotide binding protein (G protein), alpha 14 |
| GNB1L | Guanine nucleotide binding protein (G protein), beta polypeptide 1-like |
| GPC6 | Glypican 6 |
| GPR139 | G protein-coupled receptor 139 |
| GRID2 | Glutamate receptor, ionotropic, delta 2 |
| GRIN2A | Glutamate receptor, ionotropic, N-methyl D-aspartate 2A |
| GRPR | Gastrin-releasing peptide receptor |
| HDAC4 | Histone deacetylase 4 |
| HEPACAM | Hepatic and glial cell adhesion molecule |
| HNRNPH2 | Heterogeneous nuclear ribonucleoprotein H2 (H') |
| HOXA1 | Homeobox A1 |
| ICA1 | Islet cell autoantigen 1, 69kDa |
| IL1R2 | Interleukin 1 receptor, type II |
| IL1RAPL1 | Interleukin 1 receptor accessory protein-like 1 |
| IMMP2L | IMP2 inner mitochondrial membrane peptidase-like (S. cerevisiae) |
| JMJD1C | Jumonji domain containing 1C |
| KATNAL2 | Katanin p60 subunit A-like 2 |
| KCNJ10 | Potassium inwardly-rectifying channel, subfamily J, member 10 |
| KCTD13 | Potassium channel tetramerisation domain containing 13 |
| KHDRBS2 | KH domain containing, RNA binding, signal transduction associated 2 |
| KIAA1586 | KIAA1586 |
| LAMC3 | Laminin, gamma 3 |
| LRFN5 | Leucine rich repeat and fibronectin type III domain containing 5 |
| LRP2 | Low density lipoprotein receptor-related protein 2 |
| MAP2 | Microtubule-associated protein 2 |
| MAPK3 | Mitogen-activated protein kinase 3 |
| MARK1 | MAP/microtubule affinity-regulating kinase 1 |
| MBD1 | Methyl-CpG binding domain protein 1 |
| MBD4 | Methyl-CpG binding domain protein 4 |
| MBD5 | Methyl-CpG binding domain protein 5 |
| MDGA2 | MAM domain containing glycosylphosphatidylinositol anchor 2 |
| MECP2 | Methyl CpG binding protein 2 |
| MEF2C | Myocyte enhancer factor 2C |
| MYO1A | Myosin IA |
| NBEA | Neurobeachin |
| NCKAP5L | NCK-associated protein 5-like |
| NDNL2 | Necdin-like 2 |
| NF1 | Neurofibromin 1 (neurofibromatosis, von Recklinghausen disease, Watson disease) |
| NFIA | Nuclear factor I/A |
| NIPBL | Nipped-B homolog (Drosophila) |
| NLGN1 | Neuroligin 1 |
| NLGN3 | Neuroligin 3 |
| NLGN4X | Neuroligin 4, X-linked |
| NOS1AP | Nitric oxide synthase 1 (neuronal) adaptor protein |
| NRXN2 | Neurexin 2 |
| NTNG1 | Netrin G1 |
| NXPH1 | Neurexophilin 1 |
| PCDH10 | Protocadherin 10 |
| PDZD4 | PDZ domain containing 4 |
| PECR | Peroxisomal trans-2-enoyl-CoA reductase |
| PINX1 | PIN2/TERF1 interacting, telomerase inhibitor 1 |
| PLCD1 | Phospholipase C, delta 1 |
| PLN | Phospholamban |
| POGZ | Pogo transposable element with ZNF domain |
| PPP1R3F | Protein phosphatase 1, regulatory (inhibitor) subunit 3F |
| PSD3 | Pleckstrin and Sec7 domain containing 3 |
| PSMD10 | Proteasome (prosome, macropain) 26S subunit, non-ATPase, 10 |
| PTEN | Phosphatase and tensin homolog (mutated in multiple advanced cancers 1) |
| PTPN11 | Protein tyrosine phosphatase, non-receptor type 11 |
| RAB39B | RAB39B, member RAS oncogene family |
| RAI1 | Retinoic acid induced 1 |
| RBFOX1 | RNA binding protein, fox-1 homolog (C. elegans) 1 |
| RBMS3 | RNA binding motif, single stranded interacting protein 3 |
| RELN | Reelin |
| RFWD2 | Ring finger and WD repeat domain 2 |
| RGS7 | Regulator of G-protein signaling 7 |
| RIMS3 | Regulating synaptic membrane exocytosis 3 |
| ROBO1 | Roundabout, axon guidance receptor, homolog 1 (Drosophila) |
| RPS6KA2 | Ribosomal protein S6 kinase, 90kDa, polypeptide 2 |
| SATB2 | SATB homeobox 2 |
| SCN1A | Sodium channel, voltage-gated, type I, alpha subunit |
| SCN2A | Sodium channel, voltage-gated, type II, alpha subunit |
| SDC2 | Syndecan 2 (heparan sulfate proteoglycan 1, cell surface-associated, fibroglycan ) |
| SETD2 | SET domain containing 2 |
| SEZ6L2 | SEZ6L2 seizure related 6 homolog (mouse)-like 2 |
| SH3KBP1 | SH3-domain kinase binding protein 1 |
| SHANK2 | SH3 and multiple ankyrin repeat domains 2 |
| SHANK3 | SH3 and multiple ankyrin repeat domains 3 |
| SLC16A3 | Solute carrier family 16, member 3 (monocarboxylic acid transporter 4) |
| SLC30A5 | Solute carrier family 30 |
| SLC38A10 | Solute carrier family 38, member 10 |
| SLC4A10 | Solute carrier family 4, sodium bicarbonate transporter-like, member 10 |
| SLC9A6 | Solute carrier family 9 (sodium/hydrogen exchanger), member 6 |
| SLC9A9 | Solute carrier family 9 (sodium/hydrogen exchanger), member 9 |
| SNTG2 | Syntrophin, gamma 2 |
| SOX5 | SRY (sex determining region Y)-box 5 |
| SPAST | Spastin |
| ST7 | Suppression of tumorigenicity 7 |
| SUV420H1 | Suppressor of variegation 4-20 homolog 1 (Drosophila) |
| SYN1 | Synapsin 1 |
| SYNE1 | Spectrin repeat containing, nuclear envelope 1 |
| SYNGAP1 | Synaptic Ras GTPase activating protein 1 |
| TAF1L | TAF1 RNA polymerase II |
| TBC1D5 | TBC1 domain family, member 5 |
| TBL1XR1 | Transducin (beta)-like 1 X-linked receptor 1 |
| TBR1 | T-box, brain, 1 |
| TBX1 | T-box 1 |
| TGM3 | Transglutaminase 3 |
| TLK2 | Tousled-like kinase 2 |
| TMLHE | Trimethyllysine hydroxylase, epsilon |
| TLK2 | Tousled-like kinase 2 |
| TMLHE | Trimethyllysine hydroxylase, epsilon |
| TNIP2 | TNFAIP3 interacting protein 2 |
| TSC1 | Tuberous sclerosis 1 |
| TSC2 | Tuberous sclerosis 2 |
| TSPAN7 | Tetraspanin 7 |
| UBE3A | Ubiquitin protein ligase E3A |
| UBE3B | Ubiquitin protein ligase E3B |
| UBL7 | Ubiquitin-like 7 (bone marrow stromal cell-derived) |
| UBR7 | Ubiquitin protein ligase E3 component n-recognin 7 |
| UPF3B | UPF3 regulator of nonsense transcripts homolog B (yeast) |
| VPS13B | Vacuolar protein sorting 13 homolog B (yeast) |
| WNK3 | WNK lysine deficient protein kinase 3 |
| XIRP1 | Xin actin-binding repeat containing 1 |
| ZSWIM5 | Zinc finger, SWIM-type containing 5 |
|  |  |
