# Supplementary material for: Whole Genome Sequencing Reveals a De Novo SHANK3 Mutation in Familial Autism Spectrum Disorder
Source: PLoS One. 2015 Feb 3;10(2):e0116358. doi: 10.1371/journal.pone.0116358 (PMC4315573; doi:10.1371/journal.pone.0116358)
Supplement: S2 Table — (DOCX) [file pone.0116358.s004.docx]

**S2 TABLE - INHERITANCE MODELS**

**Recessive Model: Homozygous variants**

| **Gene Name** | **Chr** | **Position** | **rsSNP ID** | **Reference Allele** | **Alternate Allele** | **Effect** | **Impact** | **Frequency (%; dbSNP, ESP)** | **Present in ASD List?** | **Comments** |
| --- | --- | --- | --- | --- | --- | --- | --- | --- | --- | --- |
| **61E3.4** | 16 | 29395453 | rs71387660 | A | G | Missense | Moderate | - | - | - |
| **AC009060.1** | 16 | 70239325 | rs200798963 | C | T | Missense | Moderate | - | - | - |
| **AC110771.1** | 4 | 187112347 | rs146592367 | C | CGTT | Inframe Insertion | Moderate | - | - | - |
| **AGAP7** | 10 | 51465552 | rs76363375 | A | T | Missense | Moderate | - | - | - |
| **AP001468.1** | 21 | 47612867 | rs3902367 | A | G | Missense | Moderate | - | - | - |
| **C14orf23** | 14 | 29261309 | rs56025822 | A | AAAC | Inframe Insertion | Moderate | - | - | - |
| **C21orf49** | 21 | 34169317 | rs72564613; rs79751775 | C | CT | Frameshift | HIGH | - | - | - |
| **C21orf62** | 21 | 34166190 | rs74617086 | A | T | Missense | Moderate | - | - | - |
| **CABP1** | 12 | 121093631 | rs200201544 | TGC | T | Frameshift | HIGH | - | - | - |
| **CAMKK2** | 12 | 121678327 | rs63023660 | C | CTTT | Inframe Insertion | Moderate | - | - | - |
| **CCDC129** | 7 | 31697913 | rs35589779 | C | CT | Frameshift | HIGH | - | - | - |
| **CD177** | 19 | 43860192 | rs71337594 | T | G | Missense | Moderate | - | - | - |
| **CLDN16** | 3 | 190106071 | rs368234054; rs56086318 | AG | A | Frameshift | HIGH | - | - | - |
| **CLDN16** | 3 | 190106074 | rs3214506 | G | C | Missense | Moderate | - | - | - |
| **CTD-2335A18.1** | 15 | 89925828 | rs77546899 | T | TGTGA | At Splice Donor Site | HIGH | - | - | - |
| **CTD-2396E7.8** | 19 | 6562249 | rs74174810 | C | CT | At Splice Acceptor Site | HIGH | - | - | - |
| **CTD-2611O12.2** | 19 | 56284396 | rs141642534 | A | T | Missense | Moderate | - | - | - |
| **DCP1B** | 12 | 2062323 | rs149912567 | T | TTGC | Inframe Insertion with Codon Change | Moderate | - | - | - |
| **DSPP** | 4 | 88537088 | rs201754564 | A | G | Missense | Moderate | - | - | - |
| **FAM153A** | 5 | 177164044 | rs200041092 | G | A | Missense | Moderate | - | - | - |
| **FAM205A** | 9 | 34725742 | rs62547039 | T | C | Missense | Moderate | - | - | - |
| **FCGR3B** | 1 | 161599693 | rs76714703; rs448740 | T | C | Missense | Moderate | - | - | - |
| **FKSG68** | 21 | 37270507 | rs79702810 | T | C | Missense | Moderate | - | - | - |
| **FLJ22184** | 19 | 7938306 | rs58971992 | A | AG | Frameshift | HIGH | - | - | - |
| **GOLGA6L2** | 15 | 23685004 | rs34879341 | G | GCAT | Inframe Insertion | Moderate | - | - | - |
| **GOLGA6L2** | 15 | 23685541 | rs375981990 | CT | C | Frameshift | HIGH | - | - | - |
| **GOLGA6L2** | 15 | 23690543 | rs2344900 | T | C | Missense | Moderate | - | - | - |
| **GOLGA8B** | 15 | 34820269 | rs142225671 | G | A | Missense | Moderate | - | - | - |
| **GOLGA8R** | 15 | 30699639 | rs200116715 | A | C | Missense | Moderate | - | - | - |
| **HBG1** | 11 | 5270686 | rs1061234 | G | A | Missense | Moderate | - | - | - |
| **HLA-C** | 6 | 31237727 | rs2074497 | T | C | Missense | Moderate | - | - | - |
| **HLA-C** | 6 | 31237769 | rs1050105 | G | A | Missense | Moderate | - | - | - |
| **HNF1A** | 12 | 121434630 | rs55853809; rs58371019 | C | CTCATTCAT | Frameshift | HIGH | - | - | - |
| **HSH2D** | 19 | 16268207 | rs5827321 | TA | T | Frameshift | HIGH | - | - | - |
| **HSPBP1** | 19 | 55790886 | rs10701478; rs3040014 | A | AGCCGCCGCC | Inframe Insertion | Moderate | - | - | - |
| **ICA1** | 7 | 8196567 | rs200840693 | AAAAAAAAAAAAAAAAAAG | A | Inframe Deletion with Codon Change | Moderate | - | YES | mutation causing a frameshift in 1 of 12 open reading frame-containing transcripts (ENSEMBL: ENST00000407906; no data for RefSeq) |
| **KRTAP2-3** | 17 | 39216085 | rs113397060 | C | T | Missense | Moderate | - | - | - |
| **KRTAP9-6** | 17 | 39421781 | rs12938374 | A | G | Missense | Moderate | - | - | - |
| **KRTAP9-6** | 17 | 39421886 | rs12938692 | A | G | Missense | Moderate | - | - | - |
| **KRTAP9-7** | 17 | 39432017 | rs4890107 | C | T | Missense | Moderate | - | - | - |
| **LILRB3** | 19 | 54725835 | rs201948566 | G | C | Missense | Moderate | - | - | - |
| **LRRC37A2** | 17 | 44632540 | rs144051917 | T | C | Missense | Moderate | - | - | - |
| **LURAP1L** | 9 | 12775861 | rs139315731; rs3833707 | T | TGGCGGCGGC | Inframe Insertion | Moderate | - | - | - |
| **MAML2** | 11 | 95825374 | rs10557243; rs141671766 | TTGCTGCTGC | T | Inframe Deletion with Codon Change | Moderate | - | - | - |
| **MAML3** | 4 | 140651584 | rs5862430 | TCTG | T | Inframe Deletion | Moderate | - | - | - |
| **MANEA** | 6 | 96034881 | rs148967607 | A | ATATG | Frameshift | HIGH | - | - | - |
| **MAP3K4** | 6 | 161519350 | rs5881391 | CCTG | C | Inframe Deletion | Moderate | - | - | - |
| **MMP12** | 11 | 102738795 |  | G | GT | At Splice Acceptor Site | HIGH | - | - | - |
| **MPRIP** | 17 | 17039561 | rs3833098 | CCAG | C | Inframe Deletion | Moderate | - | - | - |
| **MUC4** | 3 | 195512042 | rs6805660 | T | C | Missense | Moderate | - | - | - |
| **MUC4** | 3 | 195512107 | rs374495657 | T | A | Missense | Moderate | - | - | - |
| **MUC4** | 3 | 195512186 | rs202060675 | T | C | Missense | Moderate | - | - | - |
| **MUC5B** | 11 | 1265786 | rs200197337 | C | T | Missense | Moderate | - | - | - |
| **MUC5B** | 11 | 1271321 | rs2943517 | C | G | Missense | Moderate | - | - | - |
| **NANOGP1** | 12 | 8051371 | rs61925372 | G | C | Missense | Moderate | - | - | - |
| **NBPF10** | 1 | 145360649 | rs79357135 | T | A | Missense | Moderate | - | - | - |
| **NBPF16** | 1 | 148756607 | rs373485062 | G | T | Missense | Moderate | - | - | - |
| **NCOR2** | 12 | 124824721 | rs143952466; rs61519723 | C | CGCCGCTGCT | Inframe Insertion | Moderate | - | - | - |
| **NPIPP1** | 16 | 15204289 | rs201626756 | C | T | Missense | Moderate | - | - | - |
| **OLFM1** | 9 | 137968919 | rs61043559 | G | GAA | Frameshift | HIGH | - | - | - |
| **OR2A1** | 7 | 144015434 | rs141871720 | A | G | Missense | Moderate | - | - | - |
| **OR2A1** | 7 | 144015720 | rs201568948 | C | G | Missense | Moderate | - | - | - |
| **PCDHA7** | 5 | 140214381 | rs10067182 | G | A | Missense | Moderate | - | - | - |
| **PLIN4** | 19 | 4511730 | rs62115190 | T | C | Missense | Moderate | - | - | - |
| **POTED** | 21 | 15011942 | rs200867471 | C | A | Missense | Moderate | - | - | - |
| **POTEI** | 2 | 131221170 | rs144934754 | C | T | Missense | Moderate | - | - | - |
| **PRAMEF11** | 1 | 12887549 | rs2994114 | T | C | Missense | Moderate | - | - | - |
| **PRH1** | 12 | 11035274 | rs28607516 | G | T | Missense | Moderate | - | - | - |
| **RGPD3** | 2 | 107040372 | rs200719868 | C | G | Missense | Moderate | - | - | - |
| **RP5-874C20.3** | 6 | 28239932 | rs61622742 | T | TG | At Splice Acceptor Site | HIGH | - | - | - |
| **RPSAP58** | 19 | 24010071 | rs9305011 | C | G | Missense | Moderate | - | - | - |
| **RPSAP58** | 19 | 24010781 | rs10460212 | C | T | Missense | Moderate | - | - | - |
| **SERINC2** | 1 | 31905889 | rs3050461; rs5773362 | A | ACAG | Inframe Insertion | Moderate | - | - | - |
| **SRA1** | 5 | 139931629 | rs202193903 | C | G | Missense | Moderate | - | - | - |
| **SYN2** | 3 | 12046297 | rs71624923 | A | C | Missense | Moderate | - | - | - |
| **SYN2** | 3 | 12046362 | rs2923856 | G | C | Missense | Moderate | - | - | - |
| **TMEM199** | 17 | 26708300 | rs71373645 | T | C | Missense | Moderate | - | - | - |
| **TREH** | 11 | 118529044 | rs11448549 | C | CG | Frameshift | HIGH | - | - | - |
| **TTLL3** | 3 | 9852059 | rs34761997 | C | CGAT | Inframe Insertion | Moderate | - | - | - |
| **URI1** | 19 | 30500118 | rs3840928 | GTGA | G | Inframe Deletion | Moderate | - | - | - |
| **ZFPM1** | 16 | 88599697 | rs368520732 | A | C | Missense | Moderate | - | - | - |
| **ZFPM1** | 16 | 88599698 | rs201915453 | G | C | Missense | Moderate | - | - | - |
| **ZNF717** | 3 | 75790427 | rs199946555 | C | T | Missense | Moderate | - | - | - |
| **ZNF717** | 3 | 75790811 | rs199577560 | G | T | Missense | Moderate | - | - | - |
| **ZNF98** | 19 | 22574818 | rs201005223 | T | C | Missense | Moderate | - | - | - |

**Recessive Model: Compound Heterozygous**

| **Gene Name** | **Chr** | **Position** | **rsSNP ID** | **Reference Allele** | **Alternate Allele** | **Effect** | **Impact** | **Frequency (%; dbSNP, ESP)** | **PRESENT IN ASD LIST?** |
| --- | --- | --- | --- | --- | --- | --- | --- | --- | --- |
| **AKAP3** | 12 | 4735969 | rs2041291 | G | A | Missense | Moderate | - | - |
| **AKAP3** | 12 | 4735970 | rs2041290 | A | G | Missense | Moderate | - | - |
| **ANKRD36** | 2 | 97808394 | rs62153044 | C | G | Missense | Moderate | - | - |
| **ANKRD36** | 2 | 97860487 | rs59466168 | T | C | Missense | Moderate | - | - |
| **CNTN5** | 11 | 99690286 | rs10790978 | T | G | Missense | Moderate | - | - |
| **CNTN5** | 11 | 99690428 | rs7125822 | T | G | Missense | Moderate | - | - |
| **FCGBP** | 19 | 40374034 | rs138587194 | A | G | Missense | Moderate | - | - |
| **FCGBP** | 19 | 40396150 | - | C | T | Missense | Moderate | - | - |
| **GOLGA6L2** | 15 | 23685475 | rs74189507 | C | T | Missense | Moderate | - | - |
| **GOLGA6L2** | 15 | 23685748 | rs370331313 | TCTC | T | Inframe Deletion with Codon Change | Moderate | - | - |
| **MUC3A** | 7 | 100549505 | rs74588241 | T | C | Missense | Moderate | - | - |
| **MUC3A** | 7 | 100549512 | rs77667788 | A | T | Missense | Moderate | - | - |
| **MUC3A** | 7 | 100549516 | rs76951301 | C | A | Missense | Moderate | - | - |
| **MUC3A** | 7 | 100549894 | rs78007565 | T | C | Missense | Moderate | - | - |
| **MUC3A** | 7 | 100549942 | rs73714230 | T | C | Missense | Moderate | - | - |
| **MUC3A** | 7 | 100549979 | rs73398717 | C | T | Missense | Moderate | - | - |
| **MUC3A** | 7 | 100550032 | rs111723000 | C | CCCT | Inframe Insertion | Moderate | - | - |
| **OR4C3** | 11 | 48346523 | rs72911451 | C | T | Missense | Moderate | - | - |
| **OR4C3** | 11 | 48346541 | rs75498992 | A | T | Missense | Moderate | - | - |
| **OR4C3** | 11 | 48346547 | rs79042268 | C | T | Missense | Moderate | - | - |
| **OR4C3** | 11 | 48346551 | rs77470587 | C | A | Missense | Moderate | - | - |
| **OR4C3** | 11 | 48346579 | rs72911452 | C | A | Missense | Moderate | - | - |
| **OR4C3** | 11 | 48346588 | rs75900655 | A | C | Missense | Moderate | - | - |
| **OR4C3** | 11 | 48346604 | rs75647397 | T | G | Missense | Moderate | - | - |
| **OR4C3** | 11 | 48346916 | rs77069283 | G | C | Missense | Moderate | - | - |
| **OR4C3** | 11 | 48346932 | rs80285195 | G | A | Missense | Moderate | - | - |
| **OR4C3** | 11 | 48346961 | rs75493089 | A | T | Missense | Moderate | - | - |
| **OR4C3** | 11 | 48346962 | rs74589050 | A | G | Missense | Moderate | - | - |
| **OR4C3** | 11 | 48347124 | rs79019124 | A | G | Missense | Moderate | - | - |
| **OR4C3** | 11 | 48347306 | rs73465911 | G | T | Missense | Moderate | - | - |
| **OR4C5** | 11 | 48387098 | rs77594589 | A | C | Missense | Moderate | - | - |
| **OR4C5** | 11 | 48387107 | rs77606603 | T | C | Missense | Moderate | - | - |
| **OR4C5** | 11 | 48387201 | rs77053100 | G | A | Stop Gained | HIGH | - | - |
| **OR4C5** | 11 | 48387206 | rs76817095 | T | C | Missense | Moderate | - | - |
| **OR4C5** | 11 | 48387210 | rs78428763 | C | T | Missense | Moderate | - | - |
| **OR4C5** | 11 | 48387211 | rs79985800 | C | G | Missense | Moderate | - | - |
| **OR4C5** | 11 | 48387424 | rs61917478 | G | T | Missense | Moderate | - | - |
| **OR4C5** | 11 | 48387591 | rs75297075 | A | G | Missense | Moderate | - | - |
| **OR4C5** | 11 | 48387647 | rs75615667 | A | G | Missense | Moderate | - | - |
| **OR4C5** | 11 | 48387890 | rs74623688 | T | C | Missense | Moderate | - | - |
| **OR4C5** | 11 | 48387900 | rs74338058 | G | A | Stop Gained | HIGH | - | - |
| **OR4C5** | 11 | 48387917 | rs66829866 | AT | A | Frameshift | HIGH | - | - |
| **OR4C5** | 11 | 48387960 | rs79688019 | T | C | Missense | Moderate | - | - |
| **OR8B2** | 11 | 124253161 | rs530740 | G | A | Missense | Moderate | - | - |
| **OR8B2** | 11 | 124253170 | rs530765 | G | A | Missense | Moderate | - | - |
| **OR9G1** | 11 | 56468212 | rs591369 | G | A | Missense | Moderate | - | - |
| **OR9G1** | 11 | 56468699 | rs79251113 | T | A | Missense | Moderate | - | - |
| **OR9G1** | 11 | 56468704 | rs79288825 | T | C | Missense | Moderate | - | - |
| **RP11-419C5.2** | 16 | 70010589 | rs62050636 | T | C | Missense | Moderate | - | - |
| **RP11-419C5.2** | 16 | 70010610 | rs146503764 | C | T | Missense | Moderate | - | - |
| **RP3-368B9.1** | 4 | 3589719 | rs59190481 | CA | C | Frameshift | HIGH | - | - |
| **RP3-368B9.1** | 4 | 3590823 | rs66807249; rs72180705 | GACAC | G,GAC | Frameshift | HIGH | - | - |
| **RP3-368B9.1** | 4 | 3590878 | rs62272970 | C | T | Missense | Moderate | - | - |
| **RP3-368B9.1** | 4 | 3591015 | rs62272971 | A | C | Missense | Moderate | - | - |
| **TTN** | 2 | 179394972 | - | C | CCAT | Inframe Insertion | Moderate | - | - |
| **TTN** | 2 | 179449186 | Rs72646861; SNP2-179157432 | G | A | Missense | Moderate | 0.8264, 0.1908 | - |
| **TTN** | 2 | 179455631 | rs72646845 | G | A | Missense | Moderate | 0.04591, 0.0831 | - |
| **TTN** | 2 | 179585257 | rs72648972 | G | C | Missense | Moderate | 0.1837, 0.1527 | - |
| **ZNF705G** | 8 | 7215694 | rs9693671 | A | G | Missense | Moderate | - | - |
| **ZNF705G** | 8 | 7217172 | rs56023905 | G | A | Missense | Moderate | - | - |

**X-LINKED Model**

| **Gene Name** | **Chromosome** | **Position** | **rsSNP ID** | **Reference Allele** | **Alternate Allele** | **Effect** | **Impact** | **Frequency (%; dbSNP, ESP)** | **PRESENT IN ASD LIST?** |
| --- | --- | --- | --- | --- | --- | --- | --- | --- | --- |
| **DHRSX** | X | 2139200 |  | T | C | Missense | Moderate | - | - |
| **KIAA2022** | X | 73960580 | rs138236888 | A | G | Missense | Moderate | -, 0.0284 | - |
| **LCA10** | X | 153149715 | rs6643650 | C | G | Missense | Moderate | - | - |
| **LCA10** | X | 153151277 | rs56026845 | C | G | Missense | Moderate | - | - |
| **PRDX4** | X | 23693285 | rs2109141 | C | G | Missense | Moderate | 0.786, - | - |

**High impact and rare variants**

| **Gene Name** | **Chromosome** | **Position** | **Reference Allele** | **Alternate Allele** | **Effect** | **Impact** | **PRESENT IN ASD LIST?** | **Comments** |
| --- | --- | --- | --- | --- | --- | --- | --- | --- |
| **GGA3** | 17 | 73237529 | G | GC | Frameshift | HIGH | - | - |
| **IRF2** | 4 | 185395258 | ACCT | A | At Splice Donor Site | HIGH | - | - |
| **SHANK3** | 22 | 51159471 | GC | G | Frameshift | HIGH | YES | Mutation affects the 3 transcripts reported by ENSEMBL: ENST00000414786, ENST00000262795 (NM_033517) and ENST00000445220 |
| **TMEM43** | 3 | 14172394 | AG | A | Frameshift | HIGH | - | - |
| **TXNDC8** | 9 | 113065967 | C | A | Stop Gained | HIGH | - | - |
| **ZNF488** | 10 | 48359734 | A | G | At Splice Acceptor Site | HIGH | - | - |
